# Compliance With Cannabis Act Regulations Regarding Online Promotion Among Canadian Commercial Cannabis-Licensed Firms

**DOI:** 10.1001/jamanetworkopen.2021.16551

**Published:** 2021-07-12

**Authors:** Natasha Y. Sheikhan, Ashlyn M. Pinto, Dominik A. Nowak, Farbod Abolhassani, Patrick Lefebvre, Mei Sheng Duh, Theodore J. Witek

**Affiliations:** 1 Dalla Lana School of Public Health, University of Toronto, Toronto, Ontario, Canada; 2 Department of Mental Health, Johns Hopkins Bloomberg School of Public Health, Baltimore, Maryland; 3 Department of Ophthalmology and Visual Sciences, Dalhousie University, Halifax, Nova Scotia, Canada; 4 Department of Family and Community Medicine, University of Toronto, Toronto, Ontario, Canada; 5 Groupe d’analyse, Ltée, Montréal, Québec, Canada; 6 Analysis Group Inc, Boston, Massachusetts; 7 Department of Biostatistics, Harvard T.H. Chan School of Public Health, Boston, Massachusetts

## Abstract

**Importance:**

As global jurisdictions shift toward cannabis legalization, 2 areas of public health importance relate to exposure to youth and to truthful promotion. Although Canada’s Cannabis Act specifies many prohibitions related to cannabis promotion, no systematic monitoring or enforcement among licensed firms exists. Compliance with marketing regulations has effects beyond Canadian citizens because of the global outreach of websites and social media.

**Objectives:**

To evaluate compliance among licensed firms with the Cannabis Act and analyze trends among violations regarding promotional material.

**Design, Setting, and Participants:**

This cross-sectional study evaluated cannabis-licensed firms after cannabis legalization. Data were extracted from online public platforms, including company websites, Facebook, Instagram, and Twitter, from October 1, 2019, to March 31, 2020. Descriptive statistics, Poisson regression, and logistic regression were used to analyze the associations of covariates with promotion violations.

**Main Outcomes and Measures:**

The primary outcome was characterization of type and prevalence of promotion violations. Secondary outcomes were the role of various covariates (namely, licensed firm characteristics and online platforms) in the frequency and probability of violations. Hypotheses were formulated before data collection.

**Results:**

Among 261 licensed firms, 211 (80.8%) had an online platform, including 204 (96.7%) with websites, 128 (60.7%) with Facebook, 123 (58.3%) with Instagram, and 123 (58.3%) with Twitter. Of all licensed firms with an online platform, 182 (86.3%) had at least 1 violation. Compared with websites, the risk of violations was significantly higher on Facebook (rate ratio [RR], 1.24; 95% CI, 1.11-1.39) and Instagram (RR, 1.19; 95% CI, 1.05-1.34). The most common violations included lack of age restrictions, brand glamorization, and omission of risk information. With websites as the reference group, lack of age restrictions was approximately 15 times more likely to occur on Facebook (odds ratio [OR], 14.76; 95% CI, 8.06-27.05); the odds of an age restriction violation were also higher on Instagram (OR, 2.48; 95% CI, 1.43-4.32) and Twitter (OR, 4.03; 95% CI, 2.29-7.09). For unsubstantiated claims, the odds of violations were significantly decreased on Facebook (OR, 0.23; 95% CI, 0.11-0.48) and Instagram (OR, 0.28; 95% CI, 0.14-0.57). The odds of glamorization were associated with an increase on Instagram (OR, 2.90; 95% CI, 1.72-4.88).

**Conclusions and Relevance:**

In this cross-sectional study, widespread violations were observed in online Canadian cannabis promotion. To protect public health and safety amid legalization, decision-makers should make explicit federal regulation and enforcement regarding promotional activities of cannabis retailers. These results suggest that policy and enforcement of cannabis promotion in Canada would have an international impact, from ease of access to online media and downstream consequences of unregulated promotion.

## Key Points

**Question:**

Are Canadian cannabis-licensed firms complying with the Cannabis Act when promoting online?

**Findings:**

In this cross-sectional study of 261 Canadian cannabis-licensed firms, 86.3% with an online platform had at least 1 violation, which was significantly more likely to occur on social media compared with a website. A lack of age restrictions for youth, unsubstantiated claims, omission of risk information, and glamorization of cannabis products were found.

**Meaning:**

These results suggest that stronger enforcement of the Cannabis Act that is adaptable to online environments is warranted to protect exposure to youth and ensure that consumers receive truthful and balanced promotion.

## Teaser

This cross-sectional study evaluates compliance among licensed firms in Canada to the Cannabis Act and analyzes trends among violations regarding promotional material.

## Introduction

Cannabis promotion is the subject of global health policy discourse amid jurisdictional legalization within the past decade. In 2012, Colorado and Washington were the first jurisdictions to grant licenses to regulate and promote cannabis.^1,2^ In October 2018, Canada legalized recreational cannabis under Bill C-45—the legislation that encompasses cannabis promotion.^3^ Legalization was driven by 3 federal values: protecting youth, mitigating illegal profits through criminalized sales, and protecting public health and safety.^1,3^ Legalization is widely regarded as sound social and health policy for many reasons yet comes with unique challenges. Specifically, despite legalization, it remains unclear whether cannabis promotional activities reflect the federal values. Moreover, it is unclear whether cannabis promotion is held to the same rigorous enforcement as that for other recreational substances, such as tobacco, or medical substances, such as pharmaceutical products.

Cannabis promotion comes with established public health impacts that must be managed responsibly. An increasing body of evidence suggests a causal relationship between the promotion of substances and increased use.^4,5,6^ Use has risks, including cannabis-induced psychosis and substance use disorder, among others.^7,8,9^ The historic shift from criminalization to legalization includes regulated retail and commercial production of cannabis, invoking newer principles and practices around fair balance.^10,11^ Fair balance is marketing criteria for pharmaceutical drugs, where marketers sharing benefits of a product must disclose potential risks.^11^ These criteria should also apply to cannabis because a significant proportion of retailers will also be selling and promoting medical cannabis. Cannabis legalization in Canada and the US is recent; therefore, there is a paucity of evidence to guide policy. Recent studies^12,13^ suggest an increase in frequent use after legalization and greater access to retailers. Because the long-term effects of legalization and cannabis promotion remain unclear, effective regulation is critical.

Promotion comes with unique regulatory challenges. In addition to lessons from cigarette and alcohol advertising, more recent lessons from electronic cigarettes caution against advertising explicitly and implicitly to youth through social media channels, amplified by hashtags and compensated influencers.^14,15^ To begin to address these challenges, the Cannabis Act^16^ specifies a number of prohibitions related to cannabis promotion. Nonetheless, there is no systematic monitoring or enforcement. Enforcement of marketing regulations not only affects Canadian residents but also broader populations because of the global reach of online media and an international policy example for jurisdictions considering cannabis legalization. The purpose of this study was to evaluate compliance among retail-licensed firms with the Cannabis Act and analyze promotional violation patterns, thus informing potential monitoring and enforcement by regulators.

## Methods

A cross-sectional content analysis was conducted using public domain data (retail-licensed firm websites and social media). Data from cannabis-licensed firms were extracted from October 1, 2019, to March 31, 2020. Prior content analyses of online venues for substance-related promotion reflect similar methods.^17^ The University of Toronto Health Sciences Research Ethics Board confirmed that this study, being confined to use of publicly available data and no use of humans or animals, was exempt from review. This study followed the Strengthening the Reporting of Observational Studies in Epidemiology (STROBE) reporting guideline.

### Sample Selection

Retail-licensed firms were retrieved from the Health Canada government website,^18^ including license number, license(s) granted, province, classes of cannabis authorized to sell, and date of licensing. When Health Canada did not provide the firm’s website, Google’s search engine was used to search for it. Social media pages (Facebook, Instagram, and Twitter) were included when they were hyperlinked through the firm’s website to ensure legitimacy.

All firms were screened for an active website. Through direct link from the website, active social media accounts (>1 post) and posts after cannabis legalization (October 7, 2018) were assessed. Firms did not go through the coding process if the website was down or under construction, the license was suspended, the website redirected to a US website from Health Canada’s page, or no mention of cannabis was made. Licenses were categorized into a sales license (with or without other licenses) or a nonsales license (processing, cultivation, or nursery).

### Systematic Duplicates

On occasion, Health Canada’s website listed a single licensed firm more than once. These duplicates were removed from the data set. For any firm name, platform, or province combination, a sales license superseded a nonsales license. If a firm had 2 observations with the same license (sales or nonsales), the oldest license was kept. If a company had licenses in different provinces, these were counted and analyzed separately instead of removed as duplicates.

### Coding Process and Violation Categories

A content analysis of all online venues was conducted. License category, presence of age restrictions, and the presence of terms associated with medical cannabis were abstracted from each venue. Age restrictions were determined by the presence of a mandatory age check-in for websites and a statement in their biography regarding age requirements for social media. Each social media page was screened for violations in up to 50 of their most recent posts. Licensed firms that did not have any posts after legalization were excluded from the analyses.

Before website review, a coding schema for violations was developed by 3 authors (N.Y.S., A.M.P., and T.J.W.) based on §17A-E and §18 of the Cannabis Act.^16^ Violations were further categorized based on the US Food and Drug Administration’s Office of Prescription Drug Promotion structure for informing pharmaceutical promotion in the US (Table 1). We evaluated 9 categories for coding: lack of fair balance, unsubstantiated claims, communication information about price or distribution, lack of age restrictions for youth, glamorizing the brand, depiction, endorsement, external link violation, and omission of risk information. This coding schema was piloted for the first 10 firms, reviewed for reliability, and then repeated until 50 firms were screened with no disagreements. The remaining venues were then assessed using that coding schema. Three coders collected the data (N.Y.S., A.M.P., and D.A.N.); claims were captured via screenshots for review and validation (T.J.W.), and the type of violation(s) was recorded in the master Excel data set. The number of categories violated was recorded. Of note, the number of violations per category was not evaluated (eg, the total number of unsubstantiated claims for 1 company was not recorded). Coders were assigned 10% of another coder’s data to establish interrater reliability.

**Table 1.** Codebook Violations and Subcategories as Reflected in the Cannabis Act

Code	Cannabis Act (bill C-45) excerpt^16^	Criteria
Lack of fair balance^a^	It is prohibited to promote cannabis in a manner that is likely to create an erroneous impression about its characteristics, value, quantity, composition, strength, concentration, potency, purity, quality, merit, safety, health effects or health risks [§18.A]	Cannabis benefits listed on the main page, whereas cannabis risks are less accessible
Unsubstantiated medical claim^a^	It is prohibited to promote cannabis in a manner that is false, misleading, or deceptive [§18.A]	False or misleading claims about cannabis, including lack of evidence or overstating known therapeutic benefits
Communication of information about price or distribution	Communicating information about its price or distribution [§17A]	Communicating about cost or promotions, such as competitive prices or special sales during holidays
Lack of age restrictions for youth	Reasonable grounds to believe could be appealing to young persons [§17B]	Not requiring viewers to insert their birthday for websites or not mentioning age limits in the biography for social media pages
Glamorizing the brand	By presenting it or any of its brand elements in a manner that associates it or the brand element with, or evokes a positive or negative emotion about or image of, a way of life such as one that includes glamour, recreation, excitement, vitality, risk or daring [§17F]	Selling merchandise with their product on it or slogans related to cannabis, images or videos related to social activities and leisure activities, and website designs that evoked glamour
Depiction	By means of the depiction of a person, character, or animal, whether real or fictional [§17D]	Multimedia of a person, character, or animal (eg, holding cannabis or a cannabis accessory, being in the same frame as a cannabis plant, positioned next to a claim)
Endorsement^a^	By means of a testimonial or endorsement, however displayed or communicated [§17C]	Patient, physician, or consumer testimonial regarding the company's cannabis
Omission of risk information^a^	It is prohibited to promote cannabis in a manner that is likely to create an erroneous impression about its characteristics, value, quantity, composition, strength, concentration, potency, purity, quality, merit, safety, health effects or health risks [§18.A]	Exclusion of any risk information regarding the medical use of cannabis
Violation from an external link^a^	Any violation above [§17-18A]	Link within online venue that leads to violative content in an external source

^a^ These codes were excluded if there was no claim or mention of medical cannabis on venue.

### Statistical Analysis

Descriptive statistics, a Poisson regression, and several logistic regressions were used to analyze trends among violations collected after cannabis legalization. Statistical analyses were performed with Stata software, version 14 (StataCorp LLC) using built-in regression functions. The associations were expressed as rate ratios (RRs) and odds ratios (ORs) with robust 95% CIs. Analyses were performed with 2-sided tests, and *P* < .05 was considered statistically significant.

In our regression analyses, we reshaped the data set to obtain 1 observation for each firm-platform combination. Thus, the regression data set includes 578 observations. A Poisson regression was performed to estimate the frequency of types of violations as a function of type of online platform, region, number of platforms, sales license, and license granted before October 2018. Logistic regression was performed to estimate the probability of at least 1 violation as a function of the same explanatory variables.

We also estimated the probability of at least 1 violation on a specific platform by types of violations using a logistic regression to examine the association between probability of violations on a specific platform and covariates of interest. These covariates included the region (Atlantic provinces, British Columbia, Prairies, and Quebec, with Ontario as the reference group), number of platforms, sales license, license granted before October 2018, and number of other types of violations on a platform.

## Results

### Descriptive Characteristics

Table 2 summarizes the characteristics of firms, specific violations, and platforms on which the violations occurred. A total of 261 firms across all provinces were included, after removing duplicates and suspended licenses. Retail licenses granted ranged from September 2013 to February 2020. A total of 144 firms (55.2%) included a sales license. A total of 211 firms (80.8%) had an online platform, of which 204 (96.7%) had a website, 128 (60.7%) Facebook, 123 (58.3%) Instagram, and 123 (58.3%) Twitter.

**Table 2.** Summary Statistics for License Holders Granted by Health Canada After Legalization, Aggregated by Sales and Nonsales Licenses

Variable	No. (%)		
	License		Total (N = 261)
	Sales^a^ (n = 144)	Nonsales (n = 117)	
Firms with no online platform	17 (11.8)	33 (28.2)	50 (19.2)
Firms with online platform^b^	127 (88.2)	84 (71.8)	211 (80.8)
Website	127 (100.0)	77 (91.7)	204 (96.7)
Facebook	82 (64.6)	46 (54.8)	128 (60.7)
Instagram	78 (61.4)	45 (53.6)	123 (58.3)
Twitter	78 (61.4)	45 (53.6)	123 (58.3)
Online media violations (% of license holders with online presence)			
No violations	17 (13.4)	12 (14.3)	29 (13.7)
At least 1 violation	110 (86.6)	72 (85.7)	182 (86.3)
Violations (at least 1) by platform (% of license holders with a specific platform)^c^			
Website	87 (68.5)	53 (68.8)	140 (68.6)
Facebook	73 (89.0)	40 (87.0)	113 (88.3)
Instagram	64 (82.1)	40 (88.9)	104 (84.6)
Twitter	63 (80.8)	35 (77.8)	98 (79.7)
Violation (at least 1) by type (% of license holders with online presence)^d^			
Depiction	42 (33.1)	21 (25.0)	63 (29.9)
Glamorization of brand	63 (49.6)	35 (41.7)	98 (46.4)
Omission of risk information	47 (37.0)	24 (28.6)	71 (33.6)
Promotion to those <18 y of age	81 (63.8)	60 (71.4)	141 (66.8)
Unsubstantiated claim	48 (37.8)	19 (22.6)	67 (31.8)
Other	37 (29.1)	13 (15.5)	50 (23.7)
Frequency of violations by type (% of total No. of types of violations)^e^			
Depiction	86 (14.2)	38 (11.6)	124 (13.3)
Glamorization of brand	149 (24.5)	88 (26.9)	237 (29.3)
Omission of risk information	89 (14.7)	39 (11.9)	128 (22.3)
Promotion to those <18 y of age	132 (21.7)	107 (32.7)	239 (53.7)
Unsubstantiated claim	89 (14.7)	32 (9.8)	121 (58.7)
Other	62 (10.2)	23 (7.0)	85 (100.0)

^a^ Firms with a sales license are encoded as sale (medical), sale (medical) cultivation, sale (medical) processing, or sale (medical) processing cultivation. All other firms considered as no sales license.

^b^ Total firms with online platform is the count of all firms with at least 1 online platform (ie, website, Facebook, Instagram, or Twitter).

^c^ Violations by platform conditional on whether the firm has presence on a given platform. Denominator is the number of firms present on a given platform.

^d^ Violations (at least 1) by type calculated conditional on violation occurring on any of the 4 online platforms. Denominator is total firms with online platform.

^e^ Frequency of violations by type represents the percentage of a specific type of violation of the total number of violations across all platforms.

### Violations and Venues

Patterns in violations by venue, type, and platform are listed in Table 2. A total of 182 firms (86.3%) with an online platform had at least 1 violation. The venue with the highest proportion of violations was Facebook, with 113 violoations (88.3%), followed by Instagram, with 104 violations (84.6%). The most common violations were lack of restrictions for youth, brand glamorization, and absence of risk information.

Results of the Poisson and logistic regressions are given in Table 3 (RRs and ORs, respectively). Compared with websites, the rate of violations was 24% higher on Facebook (RR, 1.24; 95% CI, 1.11-1.39); 19% higher on Instagram (RR, 1.19; 95% CI, 1.05-1.34); and 11% higher on Twitter (RR, 1.11; 95% CI, 0.98-1.27), although the latter results were not significant. There was a 3-fold increase in the odds of a violation on Facebook (OR, 3.02; 95% CI, 1.61-5.68) and a 2-fold increase on Instagram (OR, 2.15; 95% CI, 1.20-3.88), using websites as the reference group.

**Table 3.** Frequency of Types of Violations and Odds of at Least 1 Violation on a Platform^a^

Variable	Frequency of types of violations (n = 578)		At least 1 violation (n = 578)	
	Rate ratio (95% CI)	*P* value	Odds ratio (95% CI)	*P* value
Platform				
Website	1 [Reference]	NA	1 [Reference]	NA
Facebook	1.24 (1.11-1.39)	<.001	3.02 (1.61-5.68)	<.001
Instagram	1.19 (1.05-1.34)	.005	2.15 (1.20-3.88)	.01
Twitter	1.11 (0.98-1.27)	.10	1.52 (0.88-2.64)	.14
Region				
Ontario	1 [Reference]	NA	1 [Reference]	NA
Atlantic provinces^b^	1.11 (0.97-1.28)	.13	1.76 (0.76-4.06)	.18
British Columbia	1.11 (1.00-1.24)	.053	1.71 (0.98-2.97)	.06
Prairies^c^	1.08 (0.96-1.22)	.21	1.42 (0.80-2.51)	.23
Quebec	1.08 (0.88-1.33)	.47	1.47 (0.60-3.60)	.39
No. of platforms	1.05 (0.98-1.11)	.16	1.18 (0.94-1.49)	.16
Sales license	0.96 (0.87-1.05)	.36	0.82 (0.52-1.31)	.41
License awarded before October 2018	1.12 (1.01-1.24)	.03	1.75 (1.07-2.87)	.03

Abbreviation: NA, not applicable.

^a^ The rate ratios correspond to the exponential of the coefficients estimated by Poisson regression. Odds ratios correspond to the exponential of the coefficients estimated by logistic regression.

^b^ Atlantic provinces include New Brunswick, Newfoundland, Nova Scotia, and Prince Edward Island.

^c^ Prairies include Alberta, Manitoba, and Saskatchewan.

As shown in the Figure, unsubstantiated claims were associated with a 77.0% decrease in the odds of a violation on Facebook (OR, 0.23; 95% CI, 0.11-0.48), 72.0% decrease on Instagram (OR, 0.28; 95% CI, 0.14-0.57), and 65.0% decrease on Twitter (OR, 0.35; 95% CI, 0.17-0.73) compared with websites. Glamorization was associated with almost a 3-fold increase in the odds of a violation on Instagram (OR, 2.90; 95% CI, 1.72-4.88) compared with websites.

**Figure.**
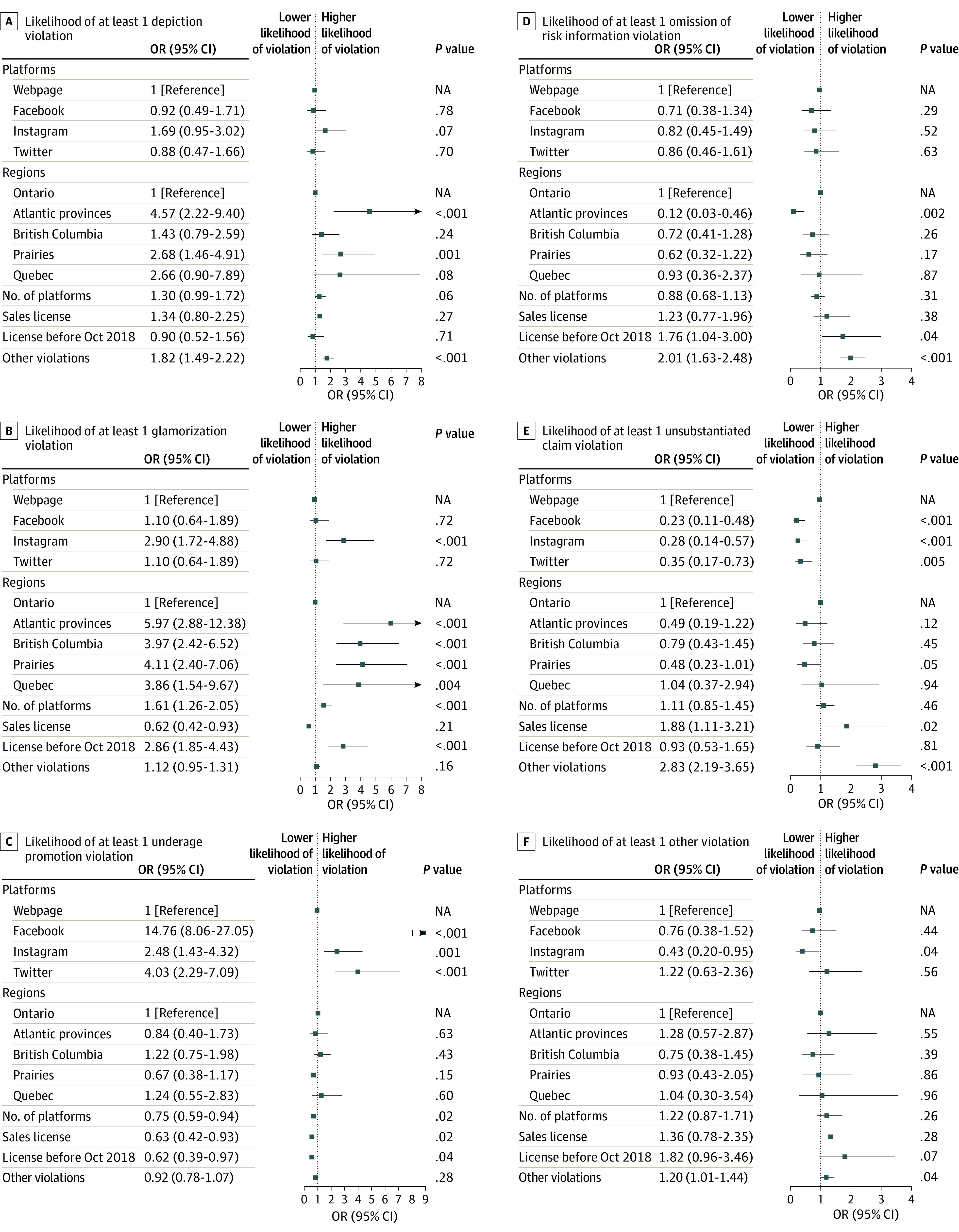
Forest Plot of the Odds Ratios (ORs) for at Least 1 Violation on a Platform by Types of Violations The ORs correspond to the exponential of the coefficients estimated by a logistic regression. Atlantic provinces include New Brunswick, Newfoundland, Nova Scotia, and Prince Edward Island. Prairies include Alberta, Manitoba, and Saskatchewan. Other violations include any violation not mentioned in the table. Error bars indicate 95% CIs. NA indicates not applicable.

### Province

In the Poisson regression, the rate of violations was not significantly different for any of the provinces when compared with Ontario (Table 3). However, as shown in the Figure, the odds of a depiction and glamorization violation were lowest with Ontario-based firms; when compared with Ontario, firms in Atlantic provinces were associated with a more than 4-fold increase in depiction violations (OR, 4.57; 95% CI, 2.22-9.40) and an almost 6-fold increase in glamorization violations (OR, 5.97; 95% CI, 2.88-12.38).

### Age Verification

Compared with websites, Facebook (OR, 14.76; 95% CI, 8.06-27.05) followed by Twitter (OR, 4.03; 95% CI, 2.29-7.09) and Instagram (OR, 2.48; 95% CI, 1.43-4.32) accounted for significant increases in the odds of a violation. The number of active platforms and whether the firm held a sales license were also significant independent variables.

### Sales License

Firms with a sales license were associated with an 88% increase in the odds of unsubstantiated claims compared with firms without a sales license (OR, 1.88; 95% CI, 1.11-3.21) (Table 3). A sales license was also associated with a 38.0% decrease in odds of glamorization (OR, 0.62; 95% CI, 0.42-0.93) and a 37.0% decrease in the odds of an age restriction violation (OR, 0.63; 95% CI, 0.42-0.93) (Figure).

### Date Granted Retail License

The rate of violations was 1.12 times higher for firms that were granted a retail license before October 2018 (RR, 1.12; 95% CI, 1.01-1.24) (Table 3). These firms also had a nearly 3-fold increase in the odds of glamorization (OR, 2.86; 95% CI, 1.85-4.43) and were associated with a 75% increase in the odds of any violation (OR, 1.75; 95% CI, 1.07-2.87) when compared with firms with licenses granted after legalization.

## Discussion

### Principal Findings

The findings of this cross-sectional study illustrate the Canadian landscape in cannabis promotion after implementation of the Cannabis Act. This study found that in general, Canadian cannabis firms are not adhering to the Cannabis Act in their promotional materials, with most having at least 1 violation. Social media platforms were especially prone to violations, in particular lack of age restrictions, risk omission, and brand glamorization.

### Relevance to the Existing Literature

Studies^19,20^ in parts of the US where recreational cannabis has been legalized corroborate these findings, reporting unsubstantiated website claims, glamorization in print media,^21^ risk omission on social media,^17^ and a lack of age restrictions.^17,20^ The findings of the current study further suggest that violations are significantly more prevalent on social media than on websites. This discrepancy raises concerns over the regulatory landscape of cannabis marketing in the US and Canada and is relevant to any jurisdiction considering cannabis promotion policy. A challenge with the cannabis industry is a dual role within the medical and recreational industries. Especially given the prevalence of unsubstantiated medical claims and omission of risk information in the current findings, cannabis advertisements with medical claims should reflect best practices in medical promotion. Pharmaceutical companies, for instance, have strict guidelines around promotion, including regulation around truthfulness and fair balance; however, medical cannabis is a product about which health claims are made but is often promoted as if it were solely recreational.^22^ Recent research^17,19,23^ has highlighted unsubstantiated claims made by cannabis companies that promote curative benefits, and researchers have advocated limiting claims to those supported by scientific evidence. Moreover, the findings of the current study reflect early promotional materials for tobacco, the perceived benefits of which were shared amid flagrant glamorization and omission of risk information.^24^ Although medical indications for cannabis exist and the science is increasing,^22,25^ firms are citing a wide range of medical uses that lack scientific validation. Unsubstantiated claims and omission of risk information do harm by preventing consumers from having a full understanding of the products.

Substances that have previously been legalized and marketed, such as tobacco and alcohol, play a role in informing cannabis health policy. Tobacco sales and use drastically increased with innovative products and marketing, which initially ignored safety concerns and targeted key populations, such as women and children.^24,26^ In the same way, the results of the current study indicate that cannabis firms promote unsubstantiated health claims, and with the internet as a platform for advertising, marketing reaches a wider audience—many of whom are youth. A recent study^14^ on JUUL (an electronic nicotine delivery system) also found that electronic cigarette marketing was accessible to youth, especially via Instagram. There was limited effectiveness in decreasing electronic cigarette–related posts on Instagram, despite the company voluntarily removing and reducing content, suggesting the need for federal interventions.^14^ Without close monitoring of cannabis promotion, the industry could follow a similar path to the tobacco industry. Specifically, the current mission to protect youth from tobacco marketing should set the precedent to protect youth from marijuana marketing.^27^

Research in alcohol marketing has found an increase in underage drinking when youths are exposed to alcohol advertisements.^26^ A study^27^ in the United Kingdom also found that Facebook is heavily used by youths and for alcohol marketing. Although Facebook requires users to be 13 years of age to create a profile, the study^27^ suggests that younger users were inputting a false birth year and accessing Facebook. These findings reflect those of the current study, which found that age restrictions were largely absent on social media. With evidence that youths are vulnerable to social media marketing, measures must be taken to regulate online cannabis marketing. Furthermore, alcohol and tobacco were historically promoted as solutions for medical ailments, but these promotions were later eliminated because of stronger regulation.^28^ Cannabis companies are currently combining recreational and medical marketing, with many glamorizing the brand. These trends are concerning and have not previously been seen in other drug and substance marketing.

### Relevance to Policy Makers

Violations were especially prevalent on social media, which is consistent with current cannabis literature. Whitehill et al^29^ found that each additional social media platform used increased past-year cannabis use in youth by 48%. Thus, regulators should evaluate cannabis firms’ adherence to promotion guidelines on all platforms but especially on social media given its marketing effectiveness with youth.

Although mechanisms exist to discourage online youth access (eg, entering birth date on websites), it is easy to falsify and difficult to regulate. The current study results revealed a lack of age restriction as the most frequent violation, which reflects previous research citing a lack of age restrictions on online promotional material for cannabis,^17,20,23^ alcohol,^30,31^ and electronic cigarettes.^15,32^ Cannabis advertising also increases intent to use among youth and perceived ease of access, which are both associated with cannabis use in adolescents.^33,34^ Although regulatory bodies prohibit promotional material to youth, the barriers to youth access are suboptimal. For instance, although Facebook has banned the promotion of cannabis sales and use, the results of this study found that most violations occurred on Facebook. This inconsistency between intended policy and real-world promotion showcases the gray areas of cannabis promotion on digital media, where exposure to youths is meant to be avoided.

A recent publication^35^ in *JAMA* highlighted the company MedMen as an example of using social media advertisements emphasizing lifestyle. Although MedMen’s marketing strategies, such as employing young models and selling products that appeal to youth, may not be intentionally targeted to youth, they indeed attract a younger population.^35^ Given that it is not necessarily legalization that increases use in youth but rather targeted promotional materials and the perception of availability, this result calls for more prudent regulation of youth-oriented advertising. Furthermore, although a recent Canadian study^36^ found that cannabis legalization did not change cannabis use patterns among high-risk youth seeking addiction services, future studies are required to examine changes among individual youths, including low-risk groups.

The results of this study suggest that current compliance with cannabis promotion regulation is suboptimal, with several observations lending themselves to policy recommendations. First, ambiguity in the Cannabis Act leads to vague recommendations and room for interpretation by firms. Rules around advertising and promotion, such as to youth, should provide clear wording on what is and is not a violation. In addition, enforcement should be in every aspect of the Cannabis Act, specifically for promoting to youth, omission of risk information, unsubstantiated claims, and diverse forms of glamorization. A potential solution could be self-regulation action as seen in other industries. Furthermore, if social media is deemed an acceptable platform to advertise cannabis, it must have clear guidelines. The addition of proactive protection against advertising targeting youth is warranted.

At a minimum, intended age limits and statements on harmful effects should be considered. In Canada, if advertising a prescription drug, only the brand name, proper name, common name, and price and quantity of the drug can be marketed.^37^ Cannabis firms that make medical claims should be held to the same standard as pharmaceutical firms; the onus should not be on the public to critically appraise medical claims. The emerging science on cannabis marketing regulation favors strict public health approaches, especially in the short term, with the potential to loosen restrictions in the future.^38,39,40^ Furthermore, promotional violations are known to only the Canadian government, the violating firm, and associated lawyers. Canada should make its monitoring of promotion and violations public to hold firms accountable, similar to the US Food and Drug Administration’s system for promotional drug violations.

### Limitations

This study has several limitations. First, many companies have multiple retail cannabis licenses under Health Canada (eg, sales, processing, and cultivation). This analysis organized licenses based on having a sales license vs not, instead of based on specific license combinations, which may have a confounding effect. Second, an assumption was made that age restrictions to enter websites or within social media pages meant that underage youth would not view or access information. However, this is based on the honor system and may lead to an underestimation of youth promotion. Third, this study only considered an omission of risk violation if it was in association with a medical claim. From a policy perspective, risks should be presented for every instance of cannabis promotion.

## Conclusions

This study offers a comprehensive analysis of cannabis promotion in Canada shortly after the federal legalization of recreational cannabis. The Cannabis Act is intended to help mitigate the risks of cannabis use, particularly those that may be exacerbated by poorly targeted or imbalanced promotion. Although cannabis legalization is a recent phenomenon with important benefits, these findings suggest that retail-licensed firms may be following similar marketing paths of alcohol, tobacco, and electronic cigarette products. The potential for widespread exposure of youths to cannabis promotion because of a lack of age restrictions, along with unsubstantiated claims, omission of risk information, and glamorization of cannabis products, is concerning. Compliance with marketing regulations likely has effects beyond Canadian citizens because of the global outreach of websites and social media, as well as relevance as a case study for other jurisdictions considering cannabis promotion policies. Greater enforcement of the Cannabis Act is crucial to protecting youth and ensuring consumers receive truthful and balanced information. The results of this study can inform future jurisdictions aiming to legalize cannabis and considering cannabis promotion policy.
