# Data Errors in a Table

**DOI:** 10.1001/jamanetworkopen.2021.25763

**Published:** 2021-08-09

In the Original Investigation titled “Compliance With Cannabis Act Regulations Regarding Online Promotion Among Canadian Commercial Cannabis-Licensed Firms,”^1^ published July 12, 2021, some percentages in Table 2, “Summary Statistics for License Holders Granted by Health Canada After Legalization, Aggregated by Sales and Nonsales Licenses,” were incorrect. Under the subheading *Frequency of violations by type (% of total No. of types of violations)*, the percentages in the last column (showing the total numbers and percentages of license holders) now read as follows: glamorization of brand, 25.4%; omission of risk information, 13.7%; promotion to those <18 y of age, 25.6%; unsubstantiated claim, 13.0%; and other, 9.1%. The total numbers were accurate, and the changes do not affect the overall conclusions of the study. The article has been corrected.^1^

## References

[1] Sheikhan NY, Pinto AM, Nowak DA, et al. Compliance with Cannabis Act regulations regarding online promotion among Canadian commercial cannabis-licensed firms. *JAMA Netw Open*. 2021;4(7):e2116551. doi:10.1001/jamanetworkopen.2021.16551 PMC8276085 34251442

